# Factors of modern lifestyle influencing gait pattern alterations in elementary school children: a mini review

**DOI:** 10.3389/fped.2026.1816590

**Published:** 2026-06-17

**Authors:** Mario Kasović, Sanja Musić Milanović, Damir Knjaz, Saša Janković, Goran Sporiš, Katarina Pavičić Dokoza, Tomáš Vespalec, Martin Sebera, Marta Gimunová, Tomislav Krističević, Goran Vrgoč, Dinko Vidović, Dario Novak, Dejan Blažević, Tihana Nemčić Bojić, Tanja Petrušič, Bruno Škrinjarić, Lenka Svobodová, Slaven Krtalić, Maja Lang Morović, Krešimir Hrg, Ana Starešinić, Bartol Vukelić, Anja Topolovec, Maja Flego Ostović, Ivana Olivari, Magda Bujan, Ljiljana Hanžek, Martina Čiček, Petra Vita Kasović, Fran Tisaj, Petra Čuljak, Mateja Očić

**Affiliations:** 1 Faculty of Kinesiology, University of Zagreb, Zagreb, Croatia; 2 Faculty of Sports Studies, Masaryk University, Brno, Czechia; 3 Croatian Institute of Public Health, Zagreb, Croatia; 4 School of Medicine, University of Zagreb, Zagreb, Croatia; 5 Orthopedic Department, Sveti Duh University Hospital, Zagreb, Croatia; 6 SUVAG Polyclinic for Hearing and Speech Rehabilitation, Zagreb, Croatia; 7 Trauma Surgery Clinic, Sestre Milosrdnice University Hospital Center, Zagreb, Croatia; 8 School of Dental Medicine, University of Zagreb, Zagreb, Croatia; 9 Institute of Sport Science and Innovations, Lithuanian Sports University, Kaunas, Lithuania; 10 School of Medicine, Catholic University of Croatia, Zagreb, Croatia Zagreb, Croatia; 11 University of Applied Health Sciences, Zagreb, Croatia; 12 Faculty of Education, University of Ljubljana, Zagreb, Croatia; 13 Institute of Economics, Zagreb, Croatia; 14 Health Center, Ozalj, Croatia; 15 August Šenoa Elementary School, Zagreb, Croatia; 16 Gustav Krklec Elementary School, Zagreb, Croatia; 17 Ivan Cankar Elementary School, Zagreb, Croatia; 18 Voltino Elementary School, Zagreb, Croatia

**Keywords:** children, elementary school, factors, gait pattern, modern lifestyle

## Abstract

Development of gait in elementary school children represents a sensitive period of locomotor maturation; however, contemporary lifestyle factors such as heavy school backpack carriage, inappropriate footwear, and the increasing prevalence of overweight and obesity can disrupt normal biomechanical gait patterns and potentially compromise musculoskeletal health. This mini review synthesizes current evidence on how selected modern lifestyle factors influence gait biomechanics in elementary school-aged children (approximately 6–14 years). Based on a comprehensive review of 35 highly relevant studies, the evidence reveals that three primary lifestyle factors significantly alter children's gait biomechanics: backpack carriage, footwear characteristics, and childhood obesity. Backpack loads exceeding 10%–12% of body weight consistently produce alterations in spatiotemporal parameters, ground reaction forces, and postural control. Footwear design, particularly sole flexibility, heel elevation, and barefoot vs. shod conditions modifies foot kinematics, plantar pressure distribution, and joint mechanics. Childhood overweight and obesity induce substantial changes in gait velocity, stride characteristics, joint loading patterns, and plantar pressures. These findings have important implications for school health policies, footwear recommendations, and obesity prevention programs. The evidence underscores the need for multifaceted interventions addressing these modifiable lifestyle factors to promote healthy musculoskeletal development and prevent long-term locomotor dysfunction in children.

## 1 Introduction

Gait patterns in children undergo substantial developmental changes throughout the elementary school years, representing a critical period for establishing mature locomotor function. However, contemporary lifestyle factors increasingly interfere with normal gait development, potentially leading to biomechanical alterations that may persist into adulthood. Elementary school children (ages 6–14 years) face unique lifestyle challenges including heavy school backpacks, inappropriate footwear, sedentary behaviours, and rising obesity rates, all of which may compromise healthy gait patterns. Understanding how these modern lifestyle factors influence pediatric gait biomechanics is essential for developing evidence-based interventions to prevent musculoskeletal disorders, optimize motor development, and promote lifelong locomotor health (1). This mini review synthesizes current research evidence on the primary lifestyle factors affecting gait patterns in elementary school children, examining the nature and magnitude of biomechanical alterations, underlying mechanisms, and implications for clinical practice and public health policy.

Mature gait patterns typically emerge by around age 7, with further refinement through adolescence. During the elementary school years, children develop stable spatiotemporal and kinematic patterns, making this period both an opportunity for optimization and a vulnerability to environmental and lifestyle influences (2–4). Gait analysis encompasses multiple domains, such as spatiotemporal parameters, kinematics, kinetics, and plantar pressure distribution (1). Alterations in any domain may affect overall locomotor efficiency and musculoskeletal loading. Contemporary lifestyle factors—including backpack loads, footwear habits, and reduced physical activity—expose elementary school children to cumulative biomechanical stress during this developmental period (5).

Although modern lifestyle encompasses a wide range of behaviours, this review focuses on three lifestyle-related factors: school backpack carriage, footwear characteristics, and childhood overweight and obesity, for which the strongest and most consistent evidence of gait-related biomechanical alterations in elementary school children currently exists. These factors are highly prevalent during this developmental period and directly impose mechanical loads on the locomotor system, resulting in reproducible changes across spatiotemporal, kinematic, kinetic, and plantar pressure domains.

## 2 Methodology of the review

This narrative mini review synthesizes evidence from a comprehensive literature search encompassing multiple databases (SciSpace, Google Scholar, PubMed) using targeted queries combining modern lifestyle factors (backpack load, footwear, obesity, physical activity, sedentary behaviour) with gait-related outcomes in elementary school-aged children. The search was conducted in January 2026 and included peer-reviewed studies published since 2000 that examined spatiotemporal, kinematic, kinetic, or plantar pressure parameters of gait in children aged approximately 6–14 years.

The initial search identified 408 records, which were merged and screened for relevance using the keywords “gait pattern,” “children,” “elementary school,” “factors,” and “modern lifestyle.” AI-powered relevance scoring was then used to prioritize studies most closely aligned with the review objectives, resulting in 118 unique articles. AI-assisted relevance scoring was used only to prioritize records, while final inclusion was determined by the authors based on relevance to the target age group, lifestyle-related exposures, and biomechanical gait outcomes. This process led to the selection of 35 studies that form the primary evidence base of this review and represent the most relevant and methodologically appropriate research addressing lifestyle-related gait alterations in the target population.

**Figure 1:**
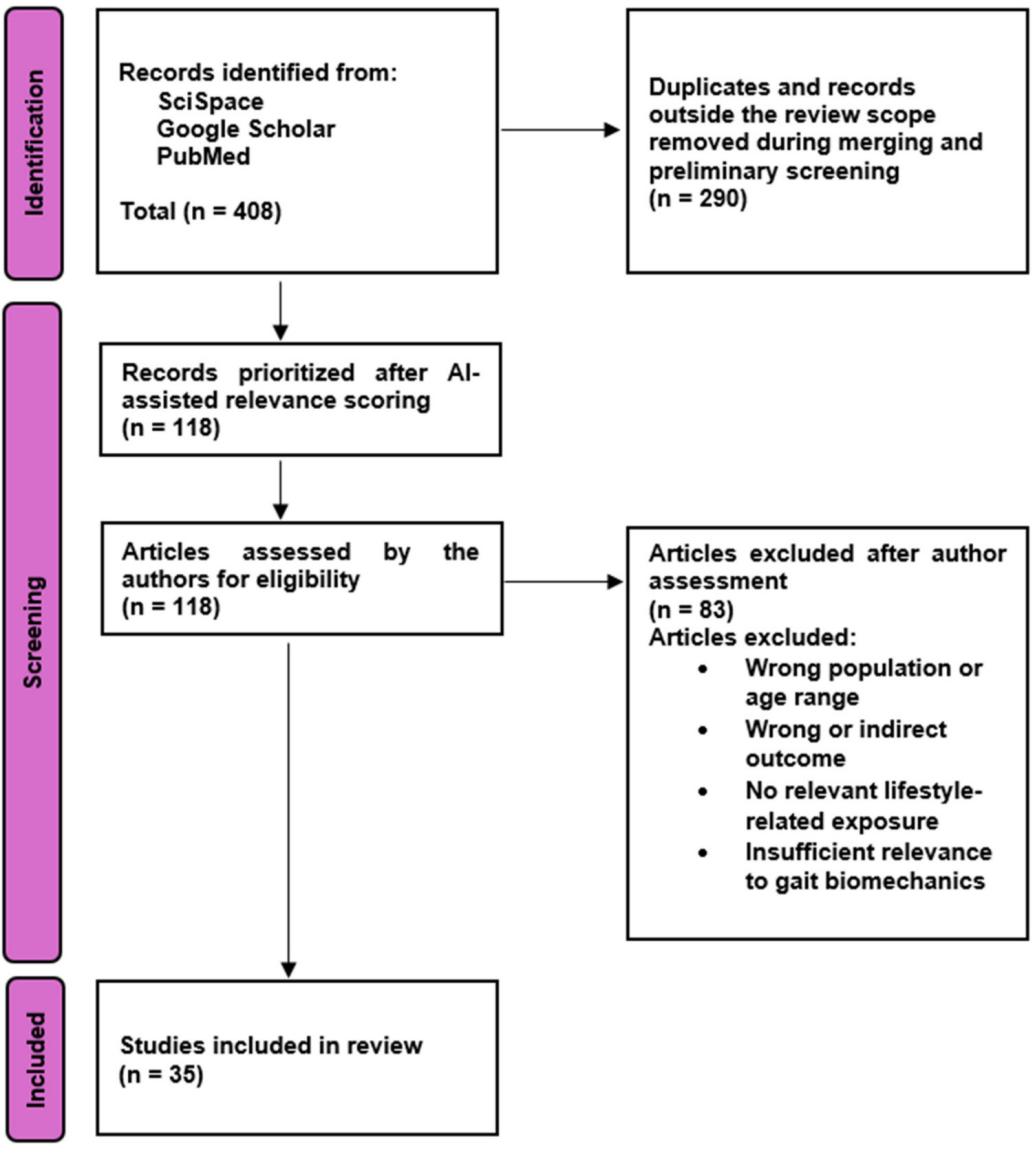
Flowchart of the literature selection process.

A narrative review was conducted due to heterogeneity in study designs and investigated factors, with the aim of identifying lifestyle factors most strongly associated with alterations in children's gait. The literature identification, prioritization, and selection process is summarized in Figure 1.

## 3 Key lifestyle factors and their effects on gait

### 3.1 Backpack load and carriage

School backpacks represent a ubiquitous lifestyle factor affecting elementary school children globally. Studies show that children often carry backpack loads ranging from 8% to 20% of body weight, frequently exceeding recommended thresholds and imposing mechanical demands on the developing locomotor system (3, 6, 7).

Backpack carriage produces systematic changes in spatiotemporal gait parameters. For example, research conducted on 85 school-going boys (ages 10–12 years) demonstrated that varying backpack loads (0%, 8%, 12%, 16%, and 20% of body weight) significantly altered the temporal patterns of peak forces during the gait cycle, with effects intensifying over walking durations up to 20 min (7). A study of 98 students aged 10–12 years using wearable inertial sensors found significant kinematic differences between free walking and walking with a backpack (6). Specific spatiotemporal changes include increased stance phase duration, reduced stride length, decreased walking velocity, and altered cadence patterns. A study of 30 children (ages 7–10 years) found that backpack loads of 10%, 15%, and 20% of body weight progressively increased stance time and double support time while decreasing swing time (3, 8). These adaptations reflect compensatory strategies to maintain stability under increased load, primarily through modifications in base of support, stance duration, and trunk posture.

Backpack carriage induces substantial alterations in joint kinematics and kinetics, including increased forward trunk lean, reduced hip and knee extension during stance, and modified ankle kinematics (9, 10), indicating a forward displacement of the center of mass and altered joint loading strategies. Changes in lower limb joint angles and electromyographic activity further suggest altered muscle recruitment strategies during loaded walking (9). Ground reaction forces are particularly sensitive to backpack loading, as progressive weight affects contact time and impact characteristics during gait (11). The temporal pattern of peak forces both the first peak (heel strike) and second peak (push-off) showed systematic alterations with increasing load and duration (7).

Backpack carriage also modifies plantar pressure patterns, particularly in overweight and obese children, suggesting additive effects of multiple risk factors and potential implications for foot pain, fatigue, and structural adaptation (3, 12).

Evidence suggests that biomechanical alterations become more pronounced when backpack loads exceed approximately 10%–12% of body weight, with 10% identified as a potential threshold for kinematic changes (8). Duration of carriage further influences these effects, as progressive alterations in peak force patterns have been observed during prolonged walking (7).

### 3.2 Footwear characteristics

Footwear represents a fundamental lifestyle factor influencing pediatric gait development. Research comparing barefoot and shod walking in children aged 6–7 years revealed significant differences in foot kinematics during both walking and running (13). Studies demonstrate that habitually barefoot children develop different foot strike patterns compared to habitually shod children, with implications for force transmission and joint loading (14). Together, these findings indicate that habitual footwear exposure shapes fundamental gait characteristics from early childhood. A systematic review and meta-analysis examining children's shoes found that footwear consistently alters gait parameters compared to barefoot walking, affecting stride length, cadence, ankle kinematics, and plantar pressure distribution (15). Research on toddlers found that children habitually wearing barefoot shoes exhibited higher plantar arches and smaller foot progression angles compared to those wearing conventional shoes, suggesting that early footwear choices may influence foot structure and function (16, 17).

Shoe flexibility emerges as a critical design parameter affecting children's gait. A systematic review specifically examining shoe flexibility found that more flexible footwear produces gait patterns more similar to barefoot walking, with reduced ankle stiffness and more natural foot motion (18). Conversely, rigid footwear may restrict natural foot mechanics, increase joint stiffness, and modify load transfer across the lower extremity. Specific footwear types produce distinct biomechanical effects. Research on thong-style flip-flops in children demonstrated altered midfoot motion during gait, with potential implications for arch development and foot stability (19). The study found that this casual footwear type, popular among children, modifies normal foot mechanics in ways that may be detrimental during critical developmental periods.

Footwear history influences fundamental gait characteristics. A study comparing children and adolescents who grew up barefoot vs. shod found significant differences in foot strike patterns, with barefoot-raised individuals more likely to exhibit forefoot or midfoot strikes compared to the rearfoot strikes typical of shod populations (14). These differences in foot strike mechanics have implications for impact forces, energy return, and lower extremity loading patterns.

### 3.3 Childhood overweight and obesity

Childhood obesity affects gait primarily by increasing mechanical load on the developing musculoskeletal system during critical developmental periods.

Overweight and obese children exhibit consistent spatiotemporal gait alterations, reflecting adaptations aimed at maintaining stability and locomotor efficiency under increased body mass. A cross-sectional study of schoolchildren found that overweight and obese children demonstrated reduced gait velocity, shorter stride length, increased stride width (wider base of support), and prolonged stance phase duration compared to normal-weight peers (20). Research on Mexican children found that overweight and obesity significantly affected biomechanical parameters of gait, with progressive alterations corresponding to increasing BMI categories (21). Longitudinal evidence suggests that persistent obesity may alter gait development trajectories during the arch development period (22). Another study examining gait smoothness in overweight (but not obese) children aged 6–10 years found subtle alterations in movement quality even at moderate levels of excess weight (23).

Obesity induces substantial kinematic alterations across multiple joints. Research examining five different walking and running velocities in overweight and obese children found consistent patterns of reduced hip and knee flexion angles, particularly during the swing phase and at initial contact (24). Biomechanical differences between obese and normal-weight children appear to persist across walking speeds, suggesting fundamental gait alterations rather than speed-related effects alone (25). A systematic review and meta-analysis reported that overweight and obese children exhibit increased stance time and altered lower limb kinematics during running and jumping, including reduced hip and knee flexion and increased hip abduction moments (5). These alterations suggest compensatory strategies for managing increased body mass and may increase cumulative joint loading over time. Obesity affects ground reaction forces and joint loading, and together with altered kinematics may increase the risk of musculoskeletal injury and early joint degeneration. Excess body mass also creates mechanical inefficiency, increases energy expenditure, and alters force distribution across joints (26). These stresses may accumulate over daily gait cycles and contribute to long-term musculoskeletal problems. Furthermore, plantar pressure distribution and foot loading patterns are influenced by obesity and may serve as early biomechanical markers of gait alterations. Higher BMI can also be accompanied by structural foot characteristics, such as a lower medial longitudinal arch or flatter foot posture, providing anatomical context for obesity-related changes in plantar pressure distribution and gait mechanics. Longitudinal evidence indicates that increasing BMI is associated with progressive arch-related changes in foot loading as children gain excess weight (27). Obese children show higher peak pressures, increased contact area, and altered pressure distribution patterns compared with normal-weight peers (28). Importantly, research demonstrated that lower activity levels in overweight children are related to higher plantar pressures, suggesting an interaction between obesity, physical activity, and biomechanical loading (5, 29). A doctoral thesis examining biomechanics of the pediatric foot and lower limb found strong associations between adiposity and altered foot mechanics, including changes in arch structure, pressure patterns, and gait characteristics (30). Obesity and backpack carriage may have additive effects on plantar pressure patterns, suggesting compounded biomechanical stress on the developing locomotor system (3, 12).

### 3.4 Emerging factors

While direct evidence on sedentary behavior and screen time effects on gait biomechanics in elementary school children remains limited in the reviewed literature, research on locomotive syndrome in school-aged children found associations between lifestyle factors and movement difficulties. Lower physical activity levels appear to interact with other factors, particularly obesity, to influence gait patterns (5, 29).

Emerging research examines how modern lifestyle behaviors such as texting while walking affect gait patterns. Although most studies focus on adults, research demonstrates that cognitive distraction during walking produces significant gait alterations, including reduced velocity, shorter stride length, and altered obstacle negotiation strategies (31). As mobile device use increases among elementary school children, cognitive–motor interference during gait represents an important area for future research.

## 4 Comparative analysis of biomechanical alterations

Children employ various compensatory strategies to manage biomechanical challenges related to their lifestyle, including increasing their base of support, prolonging double support time, and reducing walking velocity to enhance stability when carrying loads or dealing with excess weight (8, 20) (Table 1). They also modify trunk posture and alter joint angles to redistribute forces along the kinetic chain, though these adjustments may increase localized stress on certain structures (9, 10). Additionally, children often decrease stride length and adjust push-off mechanics in an effort to conserve energy under challenging conditions (24, 26). Because multiple lifestyle factors frequently coexist, their combined effects can be additive; for example, obesity and backpack carriage both contribute to changes in plantar pressure patterns, and wearing inappropriate footwear can compound biomechanical stress, indicating that interventions should target multiple factors simultaneously for maximum effectiveness (3, 12). Moreover, the elementary school years constitute a critical developmental period during which gait patterns are still maturing, and the musculoskeletal system is adapting to growth. Lifestyle influences that alter gait during this time could interfere with normal development and establish maladaptive patterns that may persist into adulthood. Longitudinal studies have shown that gait alterations related to obesity tend to evolve progressively, suggesting ongoing adaptation or potential deterioration of gait biomechanics over time (22, 27).

**Table 1:** Primary biomechanical alterations associated with major lifestyle factors.

Gait parameter	Backpack load	Inappropriate footwear	Obesity
Velocity	Decreased	Variable (depends on shoe type)	Decreased
Stride length	Decreased	Decreased (rigid shoes)	Decreased
Cadence	Variable	Increased (compensatory)	Variable
Stance time	Increased	Variable	Increased
Base of support	Increased	Variable	Increased (wider)
Hip flexion	Decreased	Variable	Decreased
Knee flexion	Decreased	Variable	Decreased
Ankle ROM	Decreased	Decreased (rigid shoes)	Variable
Trunk lean	Increased (forward)	Minimal	Increased (backward)
Peak pressure	Increased	Altered distribution	Increased
GRF magnitude	Increased	Variable	Increased

## 5 Clinical and public health implications

Evidence-based backpack guidelines for elementary school children recommend limiting loads to 10%–12% of body weight, as biomechanical alterations tend to intensify beyond this threshold (7, 8). Even appropriate loads can have cumulative effects over time, emphasizing the importance of providing children opportunities to remove backpacks during the school day (7). Proper ergonomic design features, such as padded straps, waist belts, and load distribution elements, may help mitigate some adverse effects (6, 10). Regarding footwear, shoes should allow natural foot motion, with flexible soles supporting normal ankle and midfoot mechanics during development (14, 15, 17, 18). While specific activities may require different footwear characteristics, school shoes should generally prioritize natural foot function. Preventing and addressing childhood obesity is crucial, as its associated gait alterations highlight the need for early intervention. Prevention programs should address both the musculoskeletal and metabolic consequences of excess weight while promoting physical activity to support normal gait development. Gait assessment could be integrated into pediatric health surveillance to identify at-risk children, and a multidisciplinary approach involving pediatricians, physiotherapists, exercise specialists, and nutritionists is essential for effective intervention (20, 25, 26, 29). Schools are key settings for lifestyle interventions, including backpack-load policies, physical education, footwear guidance, lockers, reduced textbook loads, and increased opportunities for physical activity. Additionally, educating children, parents, and educators about healthy gait development and lifestyle habits can foster positive behavior change (6–8, 16).

## 6 Limitations and research gaps

As this was a narrative mini review, no formal study quality or risk-of-bias assessment was conducted, and the strength of evidence should therefore be interpreted with caution. Heterogeneity in study designs, samples, gait assessment protocols, measurement systems, and outcome definitions limits direct comparison across studies and precludes quantitative synthesis. Current evidence on pediatric gait is limited by the predominance of cross-sectional study designs, restricting understanding of causality, and many studies focus on specific age groups, limiting generalizability. Variability in analysis methods complicates comparisons, and most research examines isolated factors, despite children typically being exposed to multiple lifestyle-related influences simultaneously. Significant gaps include limited data on the direct impact of sedentary behavior and screen time on gait biomechanics, few long-term studies linking childhood gait changes to adult musculoskeletal issues, and a lack of intervention research. Additionally, more precise quantification of how factors like backpack weight affect gait and improved understanding of individual susceptibility to biomechanical loading are needed. Most studies come from developed countries, with little data from diverse cultural and geographic contexts.

## 7 Future directions and recommendations

Future research on children's gait should prioritize longitudinal studies tracking development from early childhood to adolescence, intervention trials testing strategies like backpack modifications, footwear adjustments, and obesity prevention, as well as interactions among lifestyle factors affecting gait. Investigating mechanisms linking lifestyle habits to gait alterations, alongside integrating wearable sensors and machine learning for gait monitoring in natural settings, is also essential. Clinically, practitioners may consider incorporating gait assessments into routine pediatric check-ups, provide guidance on backpack safety, footwear, and physical activity, and closely monitor children at higher risk, such as those with obesity or heavy loads. Policy measures should aim to limit backpack weights to approximately 10%–12% of body weight, enhance physical education programs, promote appropriate footwear through evidence-based guidelines, and support comprehensive obesity prevention strategies. Broader public health initiatives should raise awareness about lifestyle impacts on gait, design communities that encourage active transportation, and establish surveillance systems to monitor gait health trends and evaluate intervention effectiveness across key developmental stages.

## 8 Conclusion

Modern lifestyle factors substantially influence gait patterns in elementary school children, with backpack carriage, footwear characteristics, and childhood obesity emerging as primary modifiable factors affecting locomotor biomechanics. Evidence demonstrates consistent spatiotemporal, kinematic, kinetic, and plantar pressure alterations associated with these lifestyle factors, with implications for immediate comfort, injury risk, and long-term musculoskeletal health. Backpack loads exceeding approximately 10%–12% of body weight produce significant alterations in temporal force patterns, joint kinematics, and spatiotemporal parameters, with effects intensifying over time. Footwear design, particularly sole flexibility and the barefoot vs. shod distinction, modifies foot mechanics, joint loading, and overall gait characteristics during critical developmental periods. Childhood overweight and obesity induce gait alterations including reduced velocity, shortened stride length, altered joint angles, increased ground reaction forces, and modified plantar pressure patterns. These findings have implications for clinical practice, school policy, and public health. Evidence-based guidelines for backpack use, footwear selection, and obesity prevention can help mitigate adverse effects on gait development. Schools represent ideal settings for implementing interventions addressing these modifiable lifestyle factors. Future research should prioritize longitudinal studies, intervention trials, and investigations of interactions among lifestyle factors affecting gait patterns. Understanding individual variability in susceptibility to gait alterations will support more targeted interventions. Promoting healthy gait patterns in elementary school children requires a comprehensive approach involving families, schools, healthcare providers, and policymakers. Optimizing modifiable lifestyle factors during this developmental window may support healthy musculoskeletal development and lifelong mobility.
